# Pyruvate Kinase M2 Accelerates Cutaneous Wound Healing via Glycolysis and Wnt/β-Catenin Signaling

**DOI:** 10.3390/pharmaceutics15082028

**Published:** 2023-07-27

**Authors:** Eunhwan Kim, Yumi Hwang, Heejene Kim, Geon-Uk Kim, Yeong Chan Ryu, Minguen Yoon, Kang-Yell Choi

**Affiliations:** 1 Department of Biotechnology, College of Life Science and Biotechnology, Yonsei University, Seoul 03722, Republic of Korea; glowlight18@outlook.com (E.K.); ym_910@naver.com (Y.H.); heegene03@naver.com (H.K.); kwin0125@naver.com (G.-U.K.); ryc3478@naver.com (Y.C.R.); kavelon@nate.com (M.Y.); 2 CK Regeon Inc., Seoul 03722, Republic of Korea

**Keywords:** pyruvate kinase M2, glycolysis, Wnt/β-catenin signaling pathway, human keratinocytes, wound healing

## Abstract

Cutaneous wound healing is a complex and dynamic process with high energy demand. The activation of glycolysis is essential for restoring the structure and function of injured tissues in wounds. Pyruvate kinase M2 (PKM2) is an enzyme that plays a crucial role in the last step of glycolysis. PKM2-mediated glycolysis is known to play an important role in diseases related to regeneration and inflammation. However, the role of PKM2 in wound healing has not been fully elucidated. In this study, we found that PKM2 expression and pyruvate kinase (PK) activity were increased with the activation of Wnt/β-catenin signaling during wound healing in mice. TEPP-46, an allosteric activator of PKM2, enhanced HaCaT human keratinocyte migration and cutaneous wound healing with an increment of PK activity. Moreover, we confirmed the effect of co-treatment with TEPP-46 and KY19382, a Wnt/β-catenin signaling activator through the interference with the CXXC-type zinc finger protein 5 (CXXC5) Dishevelled interaction, on wound healing. The combination treatment significantly accelerated wound healing, which was confirmed by the expression level of PCNA, keratin 14, and α-SMA. Furthermore, co-treatment induced angiogenesis in the wound beds. Overall, activation of both glycolysis and Wnt/β-catenin signaling has the potential to be used as a therapeutic approach for wound healing.

## 1. Introduction

Cutaneous wound healing is a regenerative process that restores the structure and function of damaged skin tissues. The healing process occurs in four stages: hemostasis, inflammation, proliferation, and remodeling [1]. These processes are complex and dynamic, orchestrated by the interactions of various cellular compartments, such as keratinocytes, fibroblasts, endothelial cells, and immune cells [2,3,4]. Aberrant regulation of these processes results in chronic wounds and abnormal scar formation [5]. Current wound-healing treatments still have limitations. For example, growth factors have a short half-life and poor absorption rate. Antibiotic-based agents can result in cellular and organ toxicity; they show only temporary effects. For efficient wound healing, high energy was required by glycolysis for cellular processes, including proliferation, migration, and differentiation [6].

Glycolysis is a metabolic pathway that produces energy and plays a crucial role in wound healing [7]. In the last step of glycolysis, pyruvate kinase (PK) transfers the phosphate group from phosphor-enol pyruvate to ADP [8,9,10]. There are four different isoforms of PK: PKL, PKR, PKM1, and PKM2, which determine the rate of glycolysis [11,12]. Among them, pyruvate kinase M2 (PKM2) plays an important role in cell proliferation and is mainly expressed in the regenerated region of damaged tissues [10,13]. In addition, PKM2 promotes angiogenesis by facilitating endothelial cell migration and extracellular matrix (ECM) attachment [14,15]. Recent studies reported that PKM2 released by neutrophils facilitates wound healing by promoting angiogenesis [16]. However, the roles of PKM2 expression and its activity in wound healing have not yet been elucidated.

The Wnt/β-catenin signaling pathway is one of the major signaling pathways in wound healing [17]. The Wnt/β-catenin signaling pathway regulates embryonic development and adult tissue homeostasis. Moreover, the Wnt/β-catenin signaling pathway contributes to the regulation of cell proliferation, stem cell activation, and tissue regeneration during the wound-healing process [18,19,20]. In a previous study, PKM2 was characterized as a direct transcriptional target of the Wnt/β-catenin signaling pathway in colorectal cancer cells [21]. However, the relationship between PKM2 and Wnt/β-catenin signaling in wound healing has not yet been investigated. 

In this study, we investigated the role of PKM2 in the proliferation of human keratinocytes and the enhancement of cutaneous wound healing. The PKM2 level and PK activity were increased with an increment of β-catenin expression in the wound sites. The expression levels of wound-healing markers, such as proliferating cell nuclear antigen (PCNA) and keratin 14, were also increased during the wound-healing process. We confirmed that TEPP-46, a PKM2 activator, promoted the migration of human keratinocytes and wound healing in mice. Moreover, combination treatment with TEPP-46 and KY19382, a small molecule that activates Wnt/β-catenin signaling by interfering with cytosolic CXXC-type zinc finger protein 5 (CXXC5) function via CXXC5-Dishevelled interaction [22], significantly enhanced wound healing, followed by re-epithelialization and collagen deposition. PKM2 and its activity were highly increased in the wound sites by cotreatment. Furthermore, the combination treatment enhanced angiogenesis in the dermal layers of the wounds. Taken together, we suggest that dual activation of glycolysis and Wnt/β-catenin signaling is a potentially effective strategy for the treatment of cutaneous wound healing.

## 2. Materials and Methods

### 2.1. Animals 

Eight-week-old male C3H mice were purchased (Orient Bio Co., Seongnam-si, Republic of Korea). All animal experiments and procedures were approved by the Institutional Animal Care and Use Committee (IACUC) of the Yonsei Laboratory Animal Research Center (IACUC-202102-1214-01, IACUC-202104-1243-01, and IACUC-202202-1417-01). Mice were housed under an automated environmental managing system in micro-ventilated cages (Threeshine Inc., Seoul, Republic of Korea). Mice were maintained under temperature-controlled (24 °C), humidity-controlled (40–70%) and light-controlled (standard 12 h light/dark cycle) conditions and provided with food and water. The mice were raised with a chow diet and bedding (Central Lab Animal Inc., Seoul, Republic of Korea). Considering the animal use alternatives (3Rs), we performed preliminary experiments to determine the optimal concentration of TEPP-46 (Cayman Chemical, Ann Arbor, MI, USA) and KY19382 [23] on wound healing in mice. Then, we performed minimal animal experiments under optimal conditions.

### 2.2. In Vivo Wound-Healing Assay

To determine the therapeutic effects of TEPP-46 on wound healing, eight-week-old C3H mice were anesthetized, the dorsal hair was shaved using hair clippers, the skin was cleaned with 70% ethanol, and full-thickness excisional wounds (diameter = 1.0 cm) were made on the upper back of each mouse. Then, the mice were randomly separated (*n* = 6 each group), and TEPP-46 (0.5 mM), KY19382 (1 mM), or TEPP-46 and KY19382 combination, or epidermal growth factor (EGF; 100 μM, a positive control) was topically applied daily until wounds close (*n* = 6). As a negative control, one group of mice was treated with a vehicle [24]. The wounds were photographed, and their sizes were measured every other day using a caliper ruler under the assumption that the depths of wounds were constant in each mouse. Wound size reduction was calculated using the following equation: wound size reduction (%) = (At − A0)/A0 × 100, where A0 is the initial wound size, and At is the wound size at the indicated times. Mice were sacrificed using CO_2_ gas on day 7 to monitor vessel formation, and on day 14 for other tissue analyses after wounding, and the skin samples were harvested for histochemical analyses. The undersurface of the skin was photographed to detect the newly formed blood vessels.

### 2.3. Hematoxylin and Eosin (H&E) Staining 

The dissected tissues were fixed overnight in 4% neutral paraformaldehyde (PFA) and embedded in paraffin. The paraffin-embedded skin tissues were cut to a thickness of 4 μm and were attached to the slides. The slides were deparaffinized in three changes of xylene and rehydrated through a graded ethanol series. The sections were stained with hematoxylin for 5 min and with eosin for 1 min. The slides were dehydrated using a graded ethanol series, cleaned with xylene, and mounted in Permount (Fisher Scientific, Waltham, MA, USA). The H&E-stained slides were visualized using a Nikon bright-field optical microscope (TE-2000U, Nikon, Tokyo, Japan). 

### 2.4. Collagen Staining

Wound tissue sections measuring 4 μm were deparaffinized in xylene and rehydrated in ethanol before collagen staining. For picrosirius red staining, tissues were stained with Weigert’s solution for 8 min and stained with picrosirius red solution for 1 h, as previously described. The collagen fibers were stained red with blue nuclei [25].

### 2.5. Immunohistochemical Analysis

The slides were deparaffinized in xylene and rehydrated in ethanol. Antigens were retrieved by autoclaving the slides in 10 mM sodium citrate buffer (pH 6.0) for 15 min. The sections were pre-incubated in PBS and then blocked with 10% BSA in PBS for 1 h at room temperature.

For fluorescence staining, tissue sections were incubated overnight at 4 °C with primary antibodies against β-catenin (1:100; BD Transduction Laboratories, Lexington, KY, USA), PKM2 (1:800; #4053, Cell signaling Technology, Beverly, MA, USA), keratin 14 (1:100; #905304, Biolegend, San Diego, CA, USA), PCNA (1:100; Santa Cruz Technology, Dallas, TX, USA), α-smooth muscle actin (α-SMA) (1:100, #ab7817, Abcam, Cambridge, UK), or vascular endothelial growth factor A (VEGFA) (1:100; #ab52917, Abcam). The sections were washed with PBS and incubated with Alexa Fluor 488- (1:300; #A11001, Thermo Fisher Scientific, Waltham, MA, USA) or Alexa Fluor 555- (1:300; #A21428, Thermo Fisher Scientific) conjugated IgG secondary antibody for 1 h at room temperature, and counter-stained with DAPI (1:5000; #D9564, Sigma-Aldrich, St. Louis, MO, USA). The fluorescent signals were visualized using an LSM510 META confocal microscope (Carl Zeiss, Jena, Germany). 

For DAB staining, tissues were incubated with 1% H_2_O_2_ (Samchun Chemicals) for 10 min to block endogenous peroxidase activity. Before incubating the sections with mouse primary antibody, mouse IgG was blocked using a mouse-on-mouse (MOM) IgG blocking kit (Vector Laboratories, Newark, CA, USA) for 1 h. Sections were incubated with primary antibody overnight at 4 °C against CD31 (1:50, #ab28364, Abcam). After washing with PBS, the sections were incubated with biotinylated anti-rabbit (1:300, #BA-1000, Vector Laboratories) secondary antibody for 1 h at room temperature. The samples were stained with DAB (#SK-4100, Vector Laboratories) for 5–10 min and counter-stained with Mayer’s hematoxylin (#30002, Muto Pure Chemicals, Tokyo, Japan). All incubations were conducted in a humid chamber. Signals were analyzed using a bright-field microscope (Nikon TE-2000U).

### 2.6. Pyruvate Kinase (PK) Activity Assay

Tissues were lysed in 70 μL radio-immunoprecipitation assay (RIPA) buffer (Millipore, Bedford, MA, USA) for 30 min. The lysates were centrifuged at 15,000 rpm for 30 min. An equal amount of each protein was incubated with reaction buffer (100 mM KCl, 50 mM Tris, pH 7.5, 5 mM, 0.6 mM ADP, 0.5 mM phosphoenolpyruvate (PEP), 10 µM fructose-1,6-bisphosphate (FBP), 240 µM NADH, and 8 units of lactate dehydrogenase (LDH), as previously described [22]. The change in absorbance at 320 nm owing to the oxidation of NADH was measured using a FLUOstar OPTIMA luminometer (BMG Labtech, Offenburg, Germany).

### 2.7. Cell Culture

HaCaT human keratinocytes were cultured in Dulbecco’s modified Eagle’s medium (DMEM; Gibco, Grand Island, NY, USA) supplemented with 10% (*v*/*v*) heat-inactivated fetal bovine serum (FBS; Gibco), 100 mg/mL of penicillin (Gibco), and 100 mg/mL of streptomycin (Gibco), at 37 °C in a humidified incubator containing 5% (*v*/*v*) CO_2_. 

### 2.8. Cell Viability Assay

To assess cell proliferation, HaCaT human keratinocytes were seeded at a density of 5 × 10^3^ cells/well in a 96-well plate and treated with 0.1% (*v*/*v*) DMSO, TEPP-46 (0.1 or 1 μM), EGF (20 μg/mL), KY19382 (1 μM), or TEPP-46 (1 μM) and KY19382 (1 μM) combination. After treatment for 72 h, the medium was carefully replaced and incubated with 10% MTT (#M5655, Sigma-Aldrich) for 1 h at 37 °C. Formazan crystals were dissolved in a 200 μL solution of DMSO and quantified by measuring the light absorbance at 570 nm using the FLUOstar luminometer (BMG Labtech).

### 2.9. In Vitro Wound-Healing Assay

For the in vitro wound-healing assay, HaCaT human keratinocytes were seeded at a density of 4 × 10^5^ cells/well in a 12-well plate in DMEM containing 10% FBS and allowed to attach overnight. Following 24 h, cells were grown to form a monolayer and were carefully scratched with a sterile pipette tip to create a wound slit. After washing once with PBS to remove floating cells, cells were incubated with a medium containing 10% FBS with or without TEPP-46 (1 or 5 μM). As a positive control, cells were treated with EGF (20 μg/mL). After 24 h, the cells were rinsed with cold PBS, fixed, and stained with 2% crystal violet solution. The wound area was photographed using a Nikon bright-field optical microscope (Nikon). The wound closure rate was quantified using ImageJ software V1.48 (*n* = 3).

### 2.10. Statistical Analysis

Data are presented as means ± standard deviation (SD). Statistical analyses were performed using unpaired two-tailed Student’s *t*-tests. The asterisks denote statistically significant differences (* *p* < 0.05, ** *p* < 0.01, *** *p* < 0.001).

## 3. Results

### 3.1. PKM2 Is Increased during the Wound-Healing Process 

**Figure 1:**
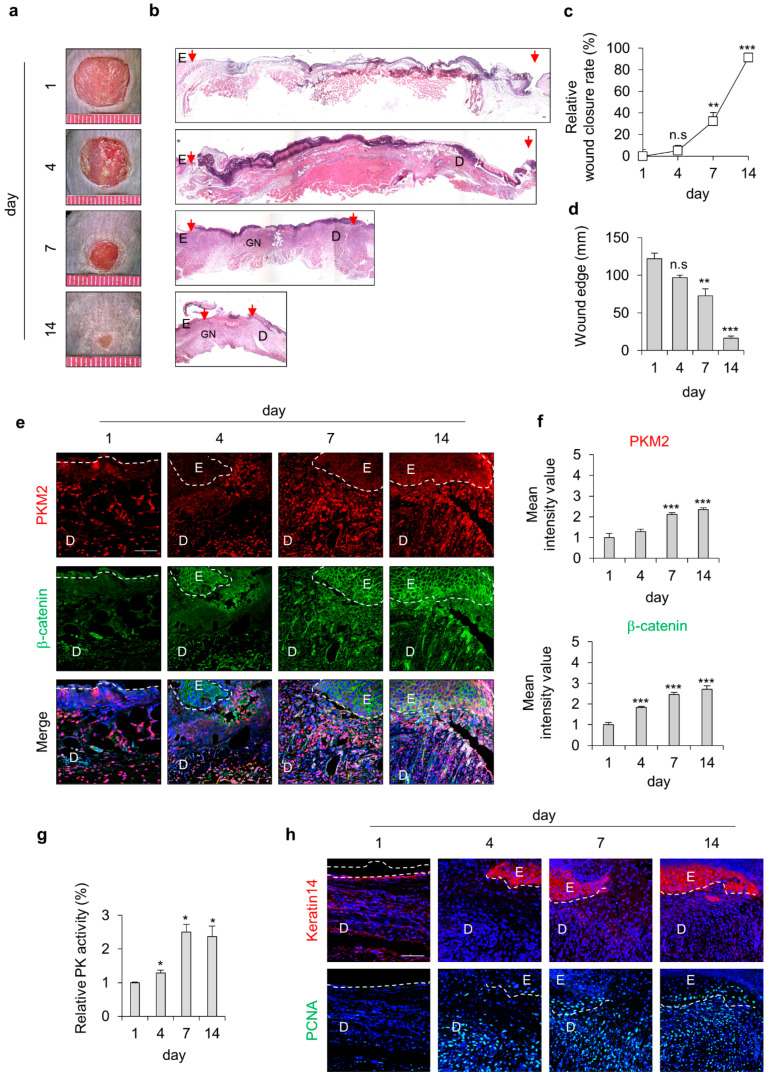
Expression profiles of PKM2 and Wnt/β-catenin signaling during the acute wound-healing process of the mouse. Full-thickness wounds were made on the back of 8-week-old male C3H mice and harvested on the indicated days, as described in the Section 2. (**a**) Representative gross images of the wounds at 1, 4, 7, and 14 days. (**b**) Representative images of H&E staining. Arrowheads indicate the wound margins; E—epidermis; D—dermis; GN—granulation tissue. (**c**) Relative wound closure rates (*n* = 6). (**d**) Quantitative analyses of wound edges (*n* = 3). (**e**) Relative images of IHC staining for PKM2 and β-catenin during the wound-healing process. Dashed lines represent the epidermal–dermal boundary. (**f**) The mean intensity value of the fluorescence signals for PKM2 and β-catenin (*n* = 3). (**g**) Relative PK activity in the wound tissues (*n* = 3). (**h**) Relative images of IHC staining for keratin 14 and PCNA. Scale bars = 100 µm. Values are expressed as mean ± SD. Student’s *t*-test (* *p* < 0.05, ** *p* < 0.01, *** *p* < 0.001, n.s.:—not significant).

To investigate the involvement of PKM2 during cutaneous wound healing, we used the mouse, acute wound-healing model. We created full-thickness wounds (diameter = 1 cm) on the backs of the mice and monitored expression levels of PKM2 during the wound-healing process (Figure 1a,b). The wound closure rates significantly increased after 4 days, and only 20% of the wound edge remained on day 14 after the wound was made (Figure 1c,d). The expression level of PKM2 was gradually increased in the wound sites, progressing to healing. The levels of β-catenin, which is essential for the proliferation of wound healing, were similarly increased in a time-dependent manner (Figure 1e,f). Moreover, we also confirmed that PK activity gradually increased and peaked after 7 days during the wound-healing process, as analyzed using tissue lysates (Figure 1g). The expression levels of keratin 14, a terminally differentiated marker of keratinocytes, and PCNA, a proliferation marker, were increased and correlated with that of PKM2 during wound healing (Figure 1h). Taken together, PKM2 expression and PK activity were increased during the proliferative phase of the wound-healing process. 

### 3.2. PKM2 Activation by TEPP-46 Enhances Migration of HaCaT Human Keratinocytes and Mouse Acute Wound Healing 

**Figure 2:**
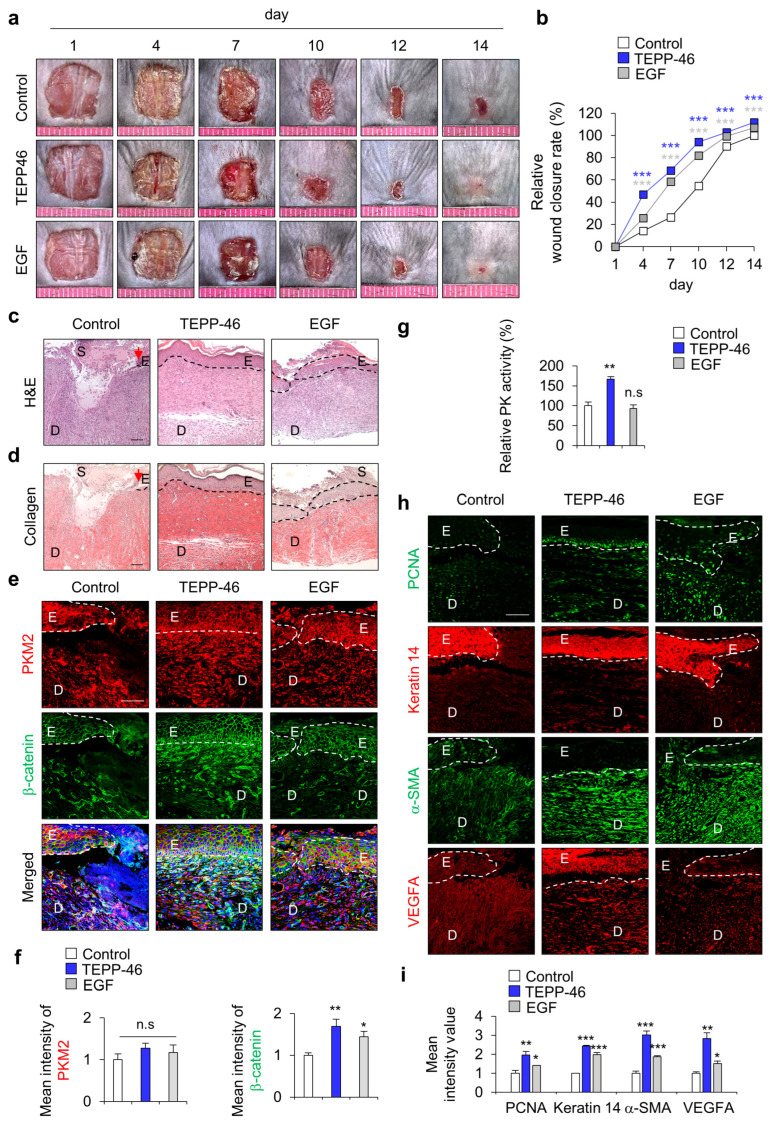
Effects of PKM2 activator TEPP-46 on wound healing in vivo. Full-thickness skin incisions (diameter = 1.0 cm) were made on the backs of 8-week-old male C3H mice, and vehicle, TEPP-46 (0.1 mM), or EGF (100 μM) was topically applied daily to the wounds for 14 days. (**a**) Representative gross images of the wounds at 1, 4, 7, 10, 12, and 14 days. (**b**) The relative wound-healing rates were measured on days 1, 4, 7, 10, and 14 days after the wounds were made and presented as the relative wound closure rate (*n* = 6). (**c**) Representative images of H&E staining. (**d**) Representative images of collagen staining with picrosirius red staining (*n* = 6). Dashed lines represent the epidermal–dermal boundary. Arrowheads indicate the wound margins; E—epidermis; D—dermis; S—scab. (**e**) Representative images of IHC staining for PKM2 and β-catenin in the wound tissues at 14 days. (**f**) Mean intensity values of the fluorescence signals for PKM2 and β-catenin in the wound tissues at 14 days. (*n* = 3). (**g**) Relative PK activity in the wound tissue lysates at 14 days (*n* = 3). (**h**) Representative images of IHC staining for PCNA, keratin 14, α-SMA, and VEGFA. (**i**) Mean intensity value of IHC staining for PCNA, keratin 14, α-SMA, and VEGFA (*n* = 3). Scale bars = 100 µm. Values are expressed as mean ± SD. Student’s *t*-test (* *p* < 0.05, ** *p* < 0.01, *** *p* < 0.001, n.s.—not significant).

To identify the effect of PKM2 activator TEPP-46 on wound healing in vivo, we used HaCaT human keratinocytes, as they play an essential role in the maintenance of skin homeostasis and re-epithelialization during the wound-healing process. We first examined the dose-dependent effects of TEPP-46 in human keratinocytes. Upon treatment with TEPP-46, the PK activity was increased in a dose-dependent manner as cell growth increased (Supplementary Figure S1a,b). Moreover, cell migration was similarly increased, and the level of increment at 1 μM was higher than that acquired with 20 μg/mL EGF (Figure S1c,d). In addition, to determine the effect of PKM2 activation on wound healing in vivo, TEPP-46 was topically applied for 14 days. Consistent with the results of in vitro experiments, the wound closure rate was increased following treatment with TEPP-46, and the degree of increment of wound closure rate was higher than that acquired by the application of EGF (Figure 2a,b). Re-epithelialization and collagen deposition, a critical component of wound healing, was increased by TEPP-46 treatment, as detected by H&E and picrosirius red collagen staining, respectively (Figure 2c,d). To investigate the role of Wnt/β-catenin signaling during the wound-healing process, we also checked the expression level of β-catenin. The expression level of β-catenin was increased, but that of PKM2 did not change significantly after TEPP-46 treatment (Figure 2e,f). The increment of the specific PK activity was confirmed only in the TEPP-46 group compared with the control group (Figure 2g). IHC analyses showed that the expression levels of both PCNA and keratin 14 were up-regulated by the TEPP-46 treatment in the neo-epidermis layers of wound tissues. Moreover, α-SMA, a marker of myofibroblast differentiation, and VEGFA, a proangiogenic factor, were increased in the wound tissues treated with TEPP-46 (Figure 2h,i). The results indicate that the promotion of wound healing by PKM2 depends on PK activity, not an increase of PKM2 expression level, during the wound-healing process. 

### 3.3. Cotreatment with Activators of Glycolysis and Wnt/β-Catenin Signaling Further Enhances Wound Healing 

**Figure 3:**
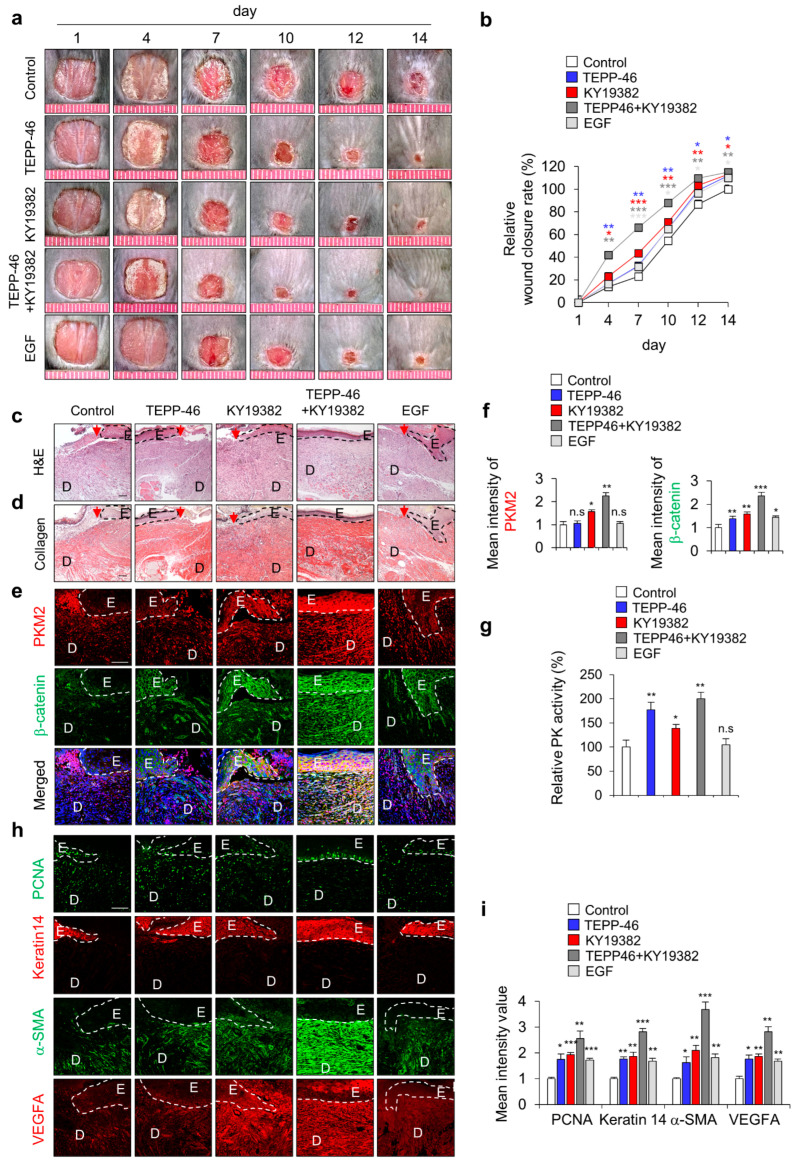
Effects of combined treatment with activators of PKM2 and Wnt/β-catenin signaling on wound healing in vivo. Full-thickness skin incisions (diameter = 1.0 cm) were made on the back of 8-week-old male C3H mice, and vehicle, TEPP-46 (0.1 mM), KY19382 (1 mM), or EGF (100 μM) were topically applied daily on the wounds for 14 days. (**a**) Representative gross images of the wounds at 1, 4, 7, 10, 12, and 14 days. (**b**) Relative wound closure rates were quantified. Wound sizes were measured at 1, 4, 7, 10, 12, and 14 days after creating wounds. (**c**,**d**) Representative images of H&E staining (upper panel) and collagen staining with picrosirius red staining (lower panel) of wound tissues (*n* = 6) at 14 days after wounds were made. (**e**) Representative images of IHC staining for PKM2 and β-catenin. (**f**) Mean intensity values of the fluorescence signals for PKM2 and β-catenin (*n* = 3). (**g**) Relative PK activity in the wound tissues at 14 days (*n* =3). (**h**) Representative images of IHC staining for PCNA, keratin 14, α-SMA, and VEGFA. (**i**) Mean intensity value of IHC staining for PCNA, keratin 14, α-SMA, and VEGFA (*n* = 3). Dashed lines represent the epidermal–dermal boundary. Arrowheads indicate the wound margins; E—epidermis; D—dermis. Scale bars = 100 µm. Values are expressed as mean ± SD. Student’s *t*-test (* *p* < 0.05, ** *p* < 0.01, *** *p* < 0.001, n.s.:—not significant).

Since we recently identified PKM2 as a direct transcriptional target of Wnt/β-catenin signaling [21], we tested the effect of combined treatment of PKM2 activator TEPP-46 and Wnt/β-catenin signaling activator KY19382 in vitro using HaCaT human keratinocytes. Co-treatment with TEPP-46 and KY19382 significantly increased PK activity as cell growth increased (Supplementary Figure S2a,b). Moreover, cell migration was also significantly increased by the combined treatment compared with each treatment (Supplementary Figure S2c,d). In addition, to further confirm the combined effect on cutaneous wound healing in vivo, we topically applied both TEPP-46 and KY19382 to the created wounds on the dorsal skin of mice (Figure 3a). As with the results of in vitro experiments, wound healing was significantly accelerated by TEPP-46 and KY19382 co-treatment on day 14, but their individual or EGF treatment still remained scab with the incomplete healing of wounds (Figure 3a,b). The combination treatment also showed a marked acceleration of re-epithelialization and collagen deposition, as determined by H&E and picrosirius collagen staining (Figure 3c,d). IHC staining showed that the expression levels of both PKM2 and β-catenin were significantly increased in the combined treatment groups. However, in the TEPP-46- or EGF-treated groups, the level of PKM2 did not change, although that of β-catenin was slightly increased (Figure 3e,f). PK activity was increased by TEPP46 and KY19382, which was further increased by co-treatment (Figure 3g). In the combined treatment of TEPP-46 and KY19382, which most significantly enhanced wound-healing rates (Figure 3a,b), both PKM2 level and its PK activity were significantly increased with the increment of β-catenin level (Figure 3e–g). Moreover, the expression levels of wound healing and angiogenesis markers, such as PCNA, keratin 14, α-SMA, and VEGFA, were also significantly enhanced by the combined treatment compared with the individual treatment of TEPP-46 or KY19382 (Figure 3h,i). Therefore, the enhancement of wound healing by the TEPP-46 and KY19382 combination treatment is attributed to both the PK activity and the increase in PKM2 level via Wnt/β-catenin signaling activation. 

### 3.4. PKM2 Promotes Angiogenesis during Wound Healing

**Figure 4:**
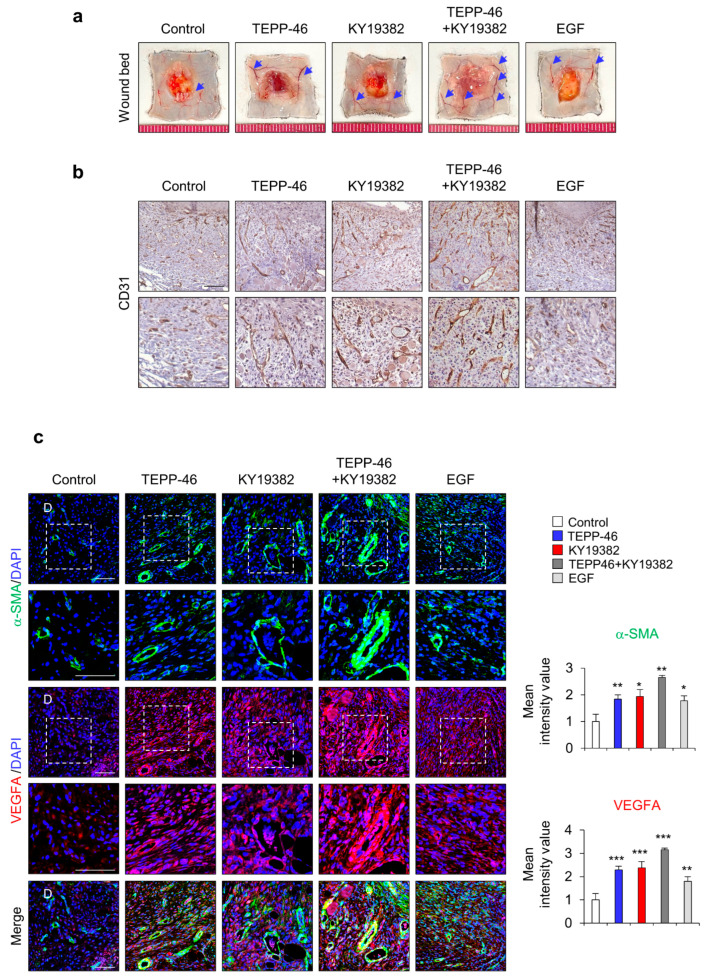
Effects of combined treatment with KY19382 and TEPP-46 on angiogenesis. Full-thickness skin incisions (diameter = 1.0 cm) were made on the back of 8-week-old male C3H mice, and vehicle, TEPP-46 (0.1 mM), KY19382 (1 mM), or EGF (100 μM) were topically applied daily on the wounds for 7 days. (**a**) Representative image of wound beds showing the development of capillaries on the cutaneous surfaces of the wound tissues. Blue arrows indicate the capillaries. (**b**) Representative images of DAB staining for CD31 in the dermis layers of the wound tissues. (**c**) Representative images of IHC staining for α-SMA and VEGFA (left panel) and mean intensity value of IHC for α-SMA and VEGFA (right panel) (*n* = 4). White dotted frame indicates lower panel as magnified images; D—dermis. Scale bars = 100 µm. Values are expressed as mean ± SD. Student’s *t*-test (* *p* < 0.05, ** *p* < 0.01, *** *p* < 0.001).

Previous studies have reported that PKM2 regulates endothelial cell junction dynamics and angiogenesis [26,27]; we checked angiogenesis status by monitoring the development of blood vessels and the angiogenesis marker CD31 in the wound sites on day 7 after the wounds were made. The blood vessels on the subcutaneous surface of the wounds were significantly increased by the combined treatment, although individual treatment with TEPP-46 or KY19382 slightly increased angiogenesis (Figure 4a). The increment of angiogenesis was further confirmed by IHC detection of CD31 expression. CD31^+^ capillary density was significantly increased by TEPP-46 and KY19382 combination treatment (Figure 4b). Furthermore, angiogenesis accompanying microvascular density was also highly increased in the combined treatment group, as shown by IHC staining for α-SMA and VEGFA (Figure 4c). Taken together, combined treatment with TEPP-46 and KY19382 enhanced angiogenesis with the induction of angiogenic markers, such as CD31, α-SMA and VEGFA, as well as that of wound healing itself. Therefore, PKM2 may contribute to accelerating wound healing by promoting angiogenesis.

## 4. Discussion

Cutaneous wound healing is characterized by multiple processes requiring energy for tissue repair [7,28,29]. As the medical and cosmetic markets expand, numerous wound care products have been developed as agents for wound healing. However, they still have limitations because of their high cost and poor efficacy [30]. Antimicrobial agents have been widely used to prevent infection of injured tissues; however, they often cause scar formation over the wound sites, which delays the wound-healing process [31]. Wet dressings, such as hydrogel dressings, maintain the moist status of wounds; however, anti-inflammatory hydrogels are still in the early stages of development. Nevertheless, they can have toxic effects in deep wounds that require sutures or in wounds with bacterial inflammation [32]. Agents using growth factors, such as EGF, are considered third-generation wound-healing agents that induce regeneration; however, they also have limitations for practical usage due to their high cost and low efficacy [33]. Therefore, a more effective therapeutic approach with new targets is required.

Wound healing depends on glycolysis for the high energy requirements for the migration and proliferation of various cells, such as keratinocytes, immune cells, epithelial cells, endothelial cells, and fibroblasts [2,3,4,7]. The activity of glycolytic enzymes is enhanced in tissues during the wound-healing process [7,34,35]. 

Glycolysis is a metabolic pathway that produces and provides energy with multiple cells involved in wound healing. Pyruvate kinases transfer the phosphate group from phosphor-enol pyruvate to ADP, which play crucial roles in the last step of glycolysis. Among the glycolytic enzymes, PKM2 is an enzyme that acts at the last stage of glycolysis [8,9,10]. The enzymatic activity of PKM2 is regulated by allosteric effects and intracellular signal transduction [36]. Previous studies of PKM2 have mainly focused on its effect on the metabolism of tumor cells [37,38]. Even in non-cancerous cells, PKM2 actually provides a metabolic advantage for biosynthesis in regenerative cells, such as stem cells. [13]. Further studies are of high interest for the role of PKM2 as a key regulator of cellular pathophysiological activity in the autoimmune response and inflammatory process [39]. Wound healing and the generation of tumors share many important properties, such as the migration of macrophages, synthesis of fibroblasts, and formation of new blood vessels [40]. Therefore, the role of PKM2 in wound healing could be the ideal approach for promoting wound healing. Recent studies have shown that infiltrated neutrophils at wound sites release pyruvate kinase M2 (PKM2), promoting wound healing [16]. PKM2 also mediates fibroblast proliferation *n* renal interstitial fibrosis [41]. 

The Wnt/β-catenin signaling pathway is essential for embryo development, adult tissue homeostasis, and regeneration. Aberrant regulation of the pathway is closely related to many diseases, such as hair loss, pigment disorders, wound healing, and bone diseases [42]. Recent studies have focused on the discovery strategies to evaluate the therapeutic potential of Wnt signaling activation [43]. The Wnt/β-catenin signaling pathway is also one of the major signaling pathways known to be involved in wound healing and tissue regeneration [17]. Moreover, activation of the Wnt/β-catenin signaling pathway induces multiple genes involving wound healing, such as *collagen 1*, *keratin 14*, *Fibronectin*, and *Endothelin 1* [18,19,20]. In a previous study, PKM2 was characterized as a direct transcriptional target of the Wnt/β-catenin signaling pathway in colorectal cancer cells [21]. However, the relationship between PKM2 and Wnt/β-catenin signaling in wound healing has not yet been investigated. Therefore, activation or an increase in the level of PKM2 could be a potential strategy for tissue repair in cutaneous wound healing. 

In this study, we investigated the role of PKM2 expression level and its activity in wound healing using the PKM2 allosteric activator TEPP-46. Especially, PKM2 level increment by KY19382-mediated Wnt/β-catenin signaling activation, as well as its activity, was investigated. Strong enhancement of wound healing by combined treatment with PKM2 and Wnt/β-catenin signaling activator compared with treatment with each TEPP-46 and KY19382 in both in vitro cell migration and in vivo wound-healing animal models. While both the level and activity of PKM2 were increased during the wound-healing process, only PK activity, but not the level, was increased by TEPP46. These results indicate that TEPP-46 enhances wound healing by the increment of PK activity. However, KY19382 enhances wound healing by increments the level of PKM2 due to it being a transcriptional target of Wnt/β-catenin signaling, as well as the expression of multiple target genes involved in wound healing. Therefore, the combined treatment of TEPP-46 and KY19382 enhances wound healing by increments of activity and level of PKM2 by TEPP-46 and KY19392, respectively, as well as by the induction of multiple target genes of the Wnt/β-catenin signaling. Taken together, our findings suggest that the activation of both glycolysis and Wnt/β-catenin signaling could be efficient therapeutic approaches for cutaneous wound healing.

## Data Availability

All data associated with this study are available from the corresponding author upon reasonable request.

## Supplementary Materials

The following supporting information can be downloaded at: https://www.mdpi.com/article/10.3390/pharmaceutics15082028/s1, Figure S1: Effects of PKM2 activator TEPP-46 on HaCaT keratinocytes in vivo; Figure S2: Effects of combined treatment with activators of PKM2 and Wnt/β-catenin signaling on wound healing in vivo.
