# Supplementary material for: Pyruvate Kinase M2 Accelerates Cutaneous Wound Healing via Glycolysis and Wnt/β-Catenin Signaling
Source: Pharmaceutics. 2023 Jul 27;15(8):2028. doi: 10.3390/pharmaceutics15082028 (PMC10458512; doi:10.3390/pharmaceutics15082028)
Supplement: Supplementary file 1 [file pharmaceutics-15-02028-s001.zip › pharmaceutics-2512719-supplementary.pdf]

Pyruvate Kinase M2 Accelerates Cutaneous Wound Healing via Glycolysis and Wnt/β-Catenin Signaling

Eunhwan Kim [1], Yumi Hwang [1], Heejene Kim [1], Geon-Uk Kim [1], Yeong Chan Ryu [1], Minguen Yoon [1] and Kang-Yell Choi [1,2,*]

[1] Department of Biotechnology, College of Life Science and Biotechnology, Yonsei University, Seoul 03722, Republic of Korea; glowlight18@outlook.com (E.K.); ym_910@naver.com (Y.H.); heegene03@naver.com (H.K.); kwin0125@naver.com (G.-U.K.); ryc3478@naver.com (Y.C.R.); kavelon@nate.com (M.Y.)

[2] CK Regeon Inc., Seoul 03722, Republic of Korea

* Correspondence: kychoi@yonsei.ac.kr

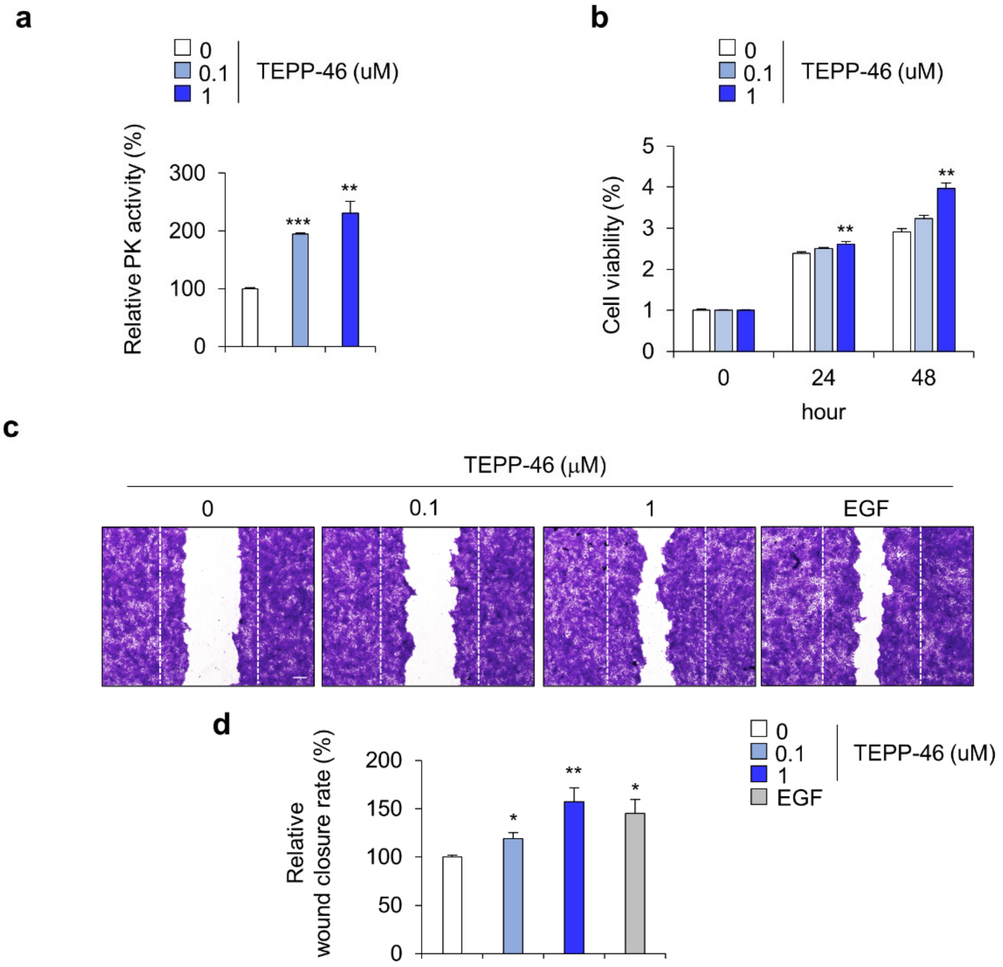

Supplementary Figure S1. Effects of PKM2 activator TEPP-46 on HaCaT keratinocytes *in vitro*.

HaCaT keratinocytes were treated with (0.1 %) DMSO, TEPP-46 (0.1 1 μM), or EGF (20 μg/ml). (**a**) Relative PK activity in HaCaT keratinocytes after TEPP-46 treatment. (**b**) Cell viability of HaCaT keratinocytes treated with the vehicle or shown concentration of TEPP-46 (*n* = 3). (**c**) HaCaT keratinocytes were treated with the vehicle, TEPP-46, or EGF for 24 h. The *in vitro* wound healing assay was performed as described in the Materials and Methods section. Representative images of the wound healing assay after crystal violet staining. (**d**) Quantitative analysis of the relative wound closure rates (*n* = 3). Scale bars = 200 μm. Values are expressed as mean ± SD. Student's t-test (* $p < 0.05$, ** $p < 0.01$, *** $p < 0.001$).

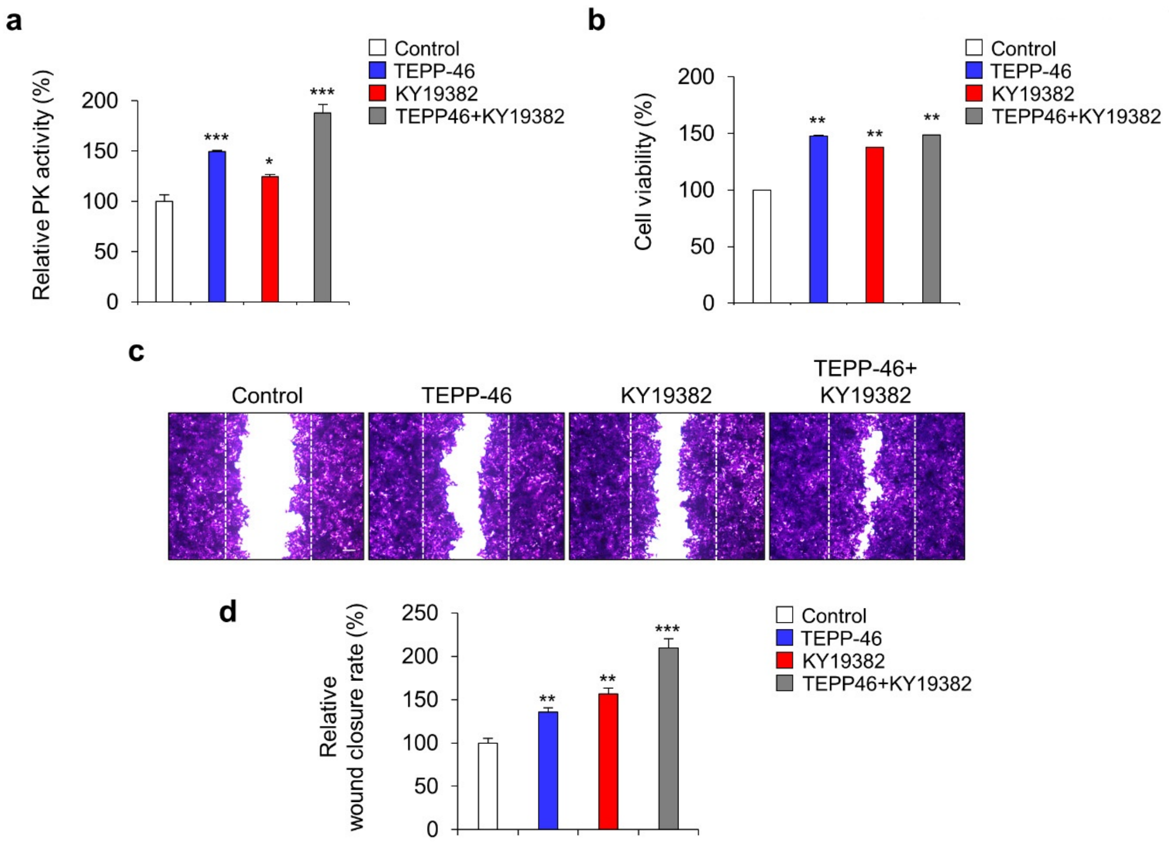

Supplementary Figure S2. Effects of combined treatment with activators of PKM2 and Wnt/β-catenin signaling on wound healing *in vitro*.

HaCaT keratinocytes were treated with (0.1 %) DMSO, TEPP-46 (1 μM), KY19382 (1 μM), or EGF (20 μg/ml). (**a**) Relative PK activity in HaCaT keratinocytes after treatment (n = 3). (**b**) Cell viability of HaCaT keratinocytes treated with the vehicle or shown concentration of TEPP-46 (n = 3). (**c**) HaCaT keratinocytes were treated with the vehicle, TEPP-46, KY19382, or EGF for 24 h. The *in vitro* wound healing assay was performed as described in the Materials and Methods section. Representative images of in vitro wound healing assay. (**d**) Quantitative analysis of relative wound closure rates. Scale bars = 200 μm. Values are expressed as mean ± SD. Student's t-test (* $p < 0.05$, ** $p < 0.01$, *** $p < 0.001$).
